# New Genome Sequences of Gamboa Viruses (Family *Bunyaviridae*, Genus *Orthobunyavirus*) Isolated in Panama and Argentina

**DOI:** 10.1128/genomeA.00940-14

**Published:** 2014-11-20

**Authors:** Marcio R. T. Nunes, Jannifer O. Chiang, Clayton P. S. de Lima, Lívia C. Martins, Amarílis Aragão Dias, Jedson F. Cardoso, Sandro P. Silva, Daisy E. A. Da Silva, Layanna F. Oliveira, Janaina M. Vasconcelos, João Paulo C. Ferreira, Amelia P. A. Travassos da Rosa, Hilda Guzman, Robert B. Tesh, Pedro F. C. Vasconcelos

**Affiliations:** a Center for Technological Innovation, Evandro Chagas Institute, Ministry of Health, Ananindeua, Brazil; b Department of Arbovirology and Hemorrhagic Fevers, Evandro Chagas Institute, Ministry of Health, Ananindeua, Brazil; c Department of Pathology, Center for Biodefense and Emerging Infectious Diseases, University of Texas Medical Branch, Galveston, Texas, USA

## Abstract

We describe here the nearly complete open reading frame (ORF) of five Gamboa virus strains isolated in Panama and Argentina. The viruses with complete ORF showed the regular genome organization observed in other orthobunyaviruses with exception to the presence of NSs protein. All predicted proteins showed homology with viruses belonging to members of the family *Bunyaviridae*.

## GENOME ANNOUNCEMENT

The family *Bunyaviridae* is one of the largest families of RNA viruses and comprises more than 350 named viruses that are currently assigned to five genera, namely, *Orthobunyavirus*, *Phlebovirus*, *Hantavirus*, *Nairovirus*, and *Tosposvirus*. Some of the viruses in the family are still ungrouped. Bunyaviruses are spherically shaped enveloped viruses with a mean diameter of 80 to 120 nm. The genome is composed of a tripartite ssRNA (SRNA, MRNA, and LRA). In general, six proteins are encoded by the three RNA segments: three are structural (Nucleocapside N and two glycoproteins Gn and Gc), and three are nonstructural (NSs, NSm, and L [polymerase]). Viruses in this family infect plants and animals; those in the genus *Tospovirus* infect plants. The vectors for these viral agents are variable and include mosquitoes, sandflies, ticks, midges, and thrips (1).

*Gamboa*-like *viruses* (GAMV) have been isolated mainly from mosquito**e**s *Aedeomyia squamipennis* in Honduras, Panama, Argentina, Ecuador, Suriname, and Brazil leading to the establishment of a new serogroup (2–4).

Five GAMV isolates were subjected to RNA extraction and nearly complete genomes were obtained using a combination of two next generation platforms (GSFLX 454 and Ion PGM torrent) (5, 6) Among four isolated from Panama *Gamboa virus* strain GML 903023 has it genome completed with segments S, M, and L, respectively, with 889; 4,988; and 6,944 nt. The *Gamboa virus* strain GML 435718 (S = 919; M = 5,002; and L = 6,944 nt), the *Gamboa virus* strain MARU 10962 (S = 1,154; M = 4,919; L = 6,933); the *Alajuela virus* strain MARU 11079 (segments S, M, and L, respectively, with 1,153; 5,088; and 6,925 nt), and the *Calchaqui virus* AG 83-1347 (S = 1,175; M = 4,917; and L = 6,788) were isolated in Argentina. The *Calchaqui virus* had 62 gaps in a 3′ end open reading frame (ORF) that was masked with degenerated nucleotides (NNNN…). Regardless of the size heterogeneity of the genomes, in general, the ORFs for S, M, and L were 717 nt (geneN)/393 nt (NSs); 4,779 nt (M polyprotein); and 6,822 nt (polymerase). Sequencing steps were carried out at the Genomic Core of the Center for Technological Innovation, Evandro Chagas Institute, Brazilian Ministry of Health, Ananindeua, Brazil.

The genome was obtained employing a *de novo* hybrid assembly strategy using both Ion torrent and GS FLX 454 reads simultaneously with the software Mira version 4.0. Visual inspection was performed with the software Geneious version 6.1.4. The total genome recovered with a mean coverage of >100 fold. The six main genes N-NSs (S segment), Gn-NSm-Gc (M segment), and polymerase (L segment) were recognized. This is the first report of complete ORF genome sequences for Gamboa viruses.

### Nucleotide sequence accession numbers.

The complete genome sequence has been deposited in GenBank as follows: *Gamboa virus* strain GML 435718 (accession numbers KM272174 to KM272176), *Gamboa virus* strain GML 903023 (KM272177 to KM272179), *Gamboa virus* strain MARU 10962 (KM272180 to KM272182), *Calchaqui virus* AG 83-1347 (KM272183 to KM272185), and *Alajuela virus* strain MARU 11079 (KM272186 to KM272188).
